# *Cutibacterium modestum* and “*Propionibacterium humerusii*” represent the same species that is commonly misidentified as *Cutibacterium acnes*

**DOI:** 10.1007/s10482-021-01589-5

**Published:** 2021-05-07

**Authors:** Daniel Goldenberger, Kirstine K. Søgaard, Aline Cuénod, Helena Seth-Smith, Daniel de Menezes, Peter Vandamme, Adrian Egli

**Affiliations:** 1 Division of Clinical Bacteriology and Mycology, University Hospital Basel, University of Basel, Petersgraben 4, 4031 Basel, Switzerland; 2 Applied Microbiology Research, Department of Biomedicine, University of Basel, Basel, Switzerland; 3 Clinic of Orthopedy, Spitalzentrum Biel, Biel-Bienne, Switzerland; 4 Laboratory of Microbiology, Department of Biochemistry and Biotechnology, Faculty of Sciences, Ghent University, Ghent, Belgium; 5 BCCM/LMG Bacteria Collection, Department of Biochemistry and Biotechnology, Faculty of Sciences, Ghent University, Ghent, Belgium

**Keywords:** Clinical significance, *Cutibacterium acnes*, *Cutibacterium modestum*, Genome analysis, “*Propionibacterium humerusii*”, Taxonomy

## Abstract

*Cutibacterium* spp. play an increasing role in soft tissue and implant-associated infections. We isolated a novel *Cutibacterium* spp. from an implant and investigated this isolate using multiple identification approaches. Correct identification was hampered by inconsistent reference data. The isolate was characterised using conventional methods such as Gram stain, MALDI-TOF MS, and antimicrobial susceptibility testing against multiple antimicrobials. Partial 16S rRNA gene sequencing and whole genome sequencing were also performed. In addition, we summarised the available published sequence data and compared prior data to our strain. Conventional phenotypic identification of our isolate resulted in *Cutibacterium* spp. After analysis of 16S rRNA gene and genome sequences, our isolate was identified as *C. modestum*, a very recently described species. The 16S rRNA gene analysis was hampered by three incorrect nucleotides within the 16S rRNA gene reference sequence of *C. modestum* M12^T^ (accession no. LC466959). We also clearly demonstrate that this novel species is identical to tentatively named “*Propionibacterium humerusii*”. Retrospective data analysis indicates that *C. modestum* is a clinically important *Cutibacterium* species often misidentified as *C. acnes.* The isolation and identification of *Cutibacterium* spp. is still a challenge. The correct description of very recently named *C. modestum* and the availability of a correct 16S rRNA sequence of the type strain may help to clarify the taxonomical uncertainty concerning “*P. humerusii*”. The novel *C. modestum* is an additional, clinically important species within the genus *Cutibacterium* and may represent a new member of the human skin microbiome.

**Supplementary Information:**

The online version contains supplementary material available at 10.1007/s10482-021-01589-5.

## Introduction

In 2016, the genus *Propionibacterium* was restructured on the basis of genomic evidence, and separated into *Propionibacterium sensu stricto* and three novel genera: *Acidipropionibacterium*, *Cutibacterium,* and *Pseudopropionibacterium* (Scholz and Kilian 2016). The cutaneous species earlier classified as *Propionibacterium acnes*, *P. avidum*, *P. granulosum,* and *P. namnetense* (Aubin et al. 2016) were assigned to the new genus *Cutibacterium.* While *C. acnes* is well-known for its potential to cause acne vulgaris, post-surgical infections and other human infections, less is known for the other species. In 2011, a novel *Propionibacterium* species was reported in a patient with humeral infection after revision of a total shoulder arthroplasty. This new species was tentatively named “*P. humerusii*” based on genomic data (Butler-Wu et al. 2011). Very recently, a Japanese group described an isolate from a patient with inflamed meibomian glands for which they formally proposed the name *Cutibacterium modestum* sp. nov. (Dekio et al. 2020).

Here, we describe a novel clinical isolate belonging to *Cutibacterium modestum* from a patient with an infected hip implant. During our analyses of this strain and retrospective data analysis of similar 16S rRNA gene and whole genome sequences, we found evidence that “*P. humerusii*” and *C. modestum* represent the same species and that this species is often misidentified as *C. acnes*.

## Material and methods

### Culture, identification methods, and antimicrobial susceptibility testing (AST)

Aerobic and anaerobic culture was performed according routine microbiological procedures. For tentative identification, we compared the obtained spectra from the MALDI-TOF MS (microflex LT, Bruker Daltonics) to the current MALDI-TOF database version (MBT 8468 MSP Library, BDAL V9.0.0.0_7854-8468). Partial 16S rRNA gene sequencing was executed as described previously (Hinic et al. 2014) and AST was performed using the gradient diffusion technology (MIC Test Strip; Liofilchem) against 20 antimicrobial agents under anaerobic conditions.

### Genome sequencing, assembly and phylogenetic analysis

**Fig. 1:**
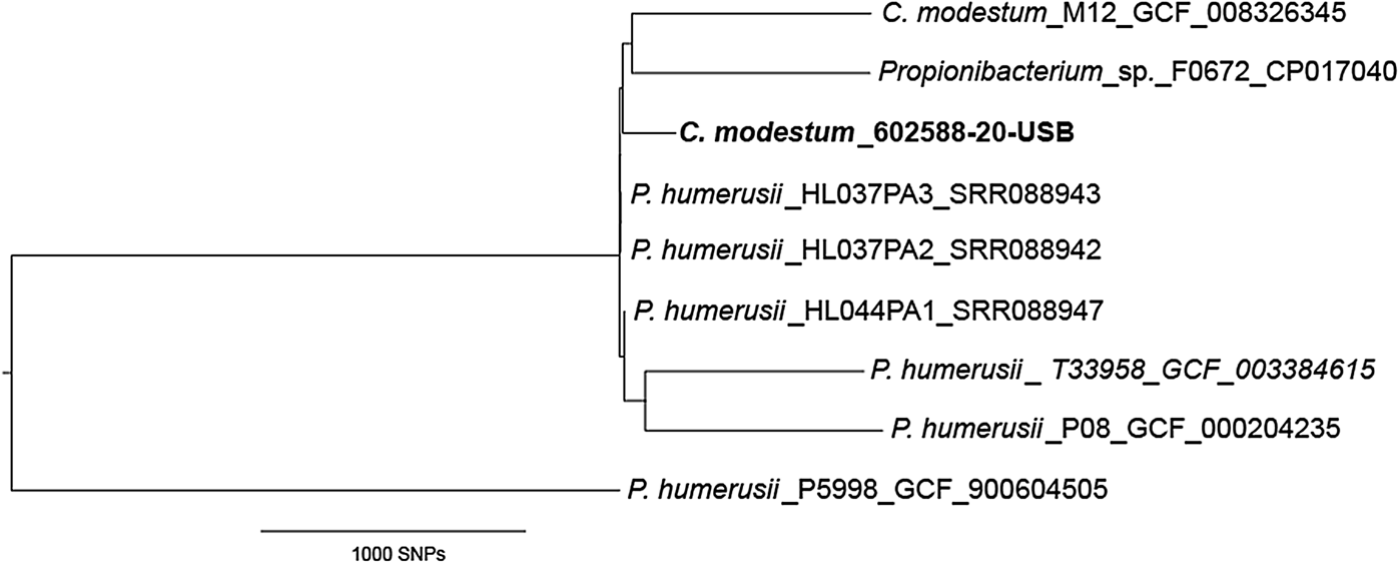
Whole genome SNP tree comparing isolates belonging to “*P. humerusii*” and *C. modestum* Neighbour joining SNP tree created using CLC genomic workbench v20.0.2. All relevant genomes were downloaded from NCBI, accession numbers are given in the figure. The newly described isolate genome is in bold. Tree rooted using the genome of the type strain of *C. acnes*: ATCC6919 (GCA_003030305, not shown). Mapping, variant calling and single nucleotide polymorphism (SNP) tree creation used parameters that differed from the default as: variant calling with single ploidy, 10 × minimum coverage, 10 minimum count and 70% minimum frequency, and SNP tree creation with 10 × minimum coverage, 10% minimum coverage, 0 prune distance and including multi-nucleotide variants (MNVs). Where required, assemblies were shredded into reads using SAMtools wgsim prior to phylogeny construction. The genome of T33958 was used as the reference (italics) as the M12 assembly is in nine contigs. All data mapped to over 97% of the reference assembly

DNA extracted from isolate 602588-20-USB was sequenced on the Illumina NextSeq platform (PE150) following library creation with Nexteraflex (Illumina). Digital DNA-DNA hybridisation (dDDH) used GGDC2.1 (http://ggdc.dsmz.de/ggdc.php#) and the DDH cut off of < 70%) (Auch et al. 2010). Reads (mean 92 × coverage) were assembled using Unicycler (Wick et al. 2017) to produce an assembly of 2.6 Mb in 22 contigs. The neighbour joining tree using whole genome SNPs was created in CLC genomic workbench v20.0.2 using parameters described in the figure legend (Fig. 1).

### Retrospective sequence analysis

Very similar 16S rRNA gene sequences and genome sequences associated with *C. modestum* were retrieved using the BLAST algorithm and were compared with the sequences from *C. modestum* strain M12^T^ and our isolate 602588-20-USB.

## Results

A 39 year old male patient presented with an implant-related hip infection following internal fixation of a femoral neck fracture. After debridement surgery and antibiotic treatment of *Staphylococcus capitis* according to susceptibility testing, no signs of persistent infection were identified at the 6 months postoperative control. *C. modestum* was not covered by antimicrobial therapy. From three cannulated screws sent for culture, growth of *Staphylococcus capitis* (> 1000 CFU/ml) and *Cutibacterium* species (80 CFU/ml) was observed. After six days of anaerobic incubation on BD Brucella blood agar (Becton Dickinson), we detected characteristic white colonies and corresponding typical Gram-positive rods that was compatible with presumptive identification of *C. acnes*. Catalase and indole reactions were positive and confirmed this suggestion. MALDI-TOF was not able to provide valid identification. The first three MALDI-TOF hits were *Propionibacterium* sp. score 1.64, *C. acnes* score 1.61 and *C. acnes* 1.51. Subsequent partial 16S rRNA gene analysis (693 bp) showed 100% identity to 9 sequences of *Propionibacterium acnes* / *Propionibacterium* spp./ “*P. humerusii*” isolates, followed by identities to type reference sequences of *C. acnes* ATCC6919 (97.7%), *C. namnetense* NTS31307302 (97.3%), *C. avidum* ATCC25577 (95.1%) *and C. granulosum* DSM20700 (93.4%). AST showed that isolate 602588-20-USB had similar patterns to other *Cutibacterium* spp., thus susceptible to common antimicrobial substances, whereas it was resistant to metronidazole and gentamicin (see supplementary materials table S1).

Retrospective analysis of 16S rRNA gene sequences in public databases resulted in 11 entries with highly similar (99.2–100%) 16S rRNA gene sequences since 2001 (excluding *C. modestum* M12^T^). The corresponding isolates originated from different countries and sources showing that this organism has a clinical relevance (Table 1), (Dekio et al. 2020; Kunishima et al. 2001; Lin et al. 2010). Five of these 11 isolates were identified as *C. acnes* despite considerable differences (97.5–98.2%) towards the 16S rRNA gene sequence of the *C. acnes* type strain (ATCC 6919^T^). We also detected within the 16S rRNA gene sequence of *C. modestum* M12^T^ (LC466959) three mismatches compared to the whole-genome-sequence of M12^T^ (BJEN01000000): position 3 a G instead of A, position 11 a T instead of C, and position 1484 an A instead of G (Dekio et al. 2020) (see also Table 1).

**Table 1:** List of probable *C. modestum* isolates (n = 13)

Strain	Specimen	Country	Year	Accession no	16S rRNA gene		References
					(bp)	(%)	
*Incorrectly labeled as Propionibacterium acnes, n* = *5*							
7375	Blood component	Japan	2001	AB042290	1482/1483	99.9	Kunishima et al. (2001)
8800	Blood component	Japan	2003	AB108481	1480/1483	99.8	unpubl
L340	“Clinical strain “	Taiwan	2010	GQ496494	1410/1421^a^	99.2	Lin et al. (2010)
NN1204	Pus	China	2015	KP944185	1418/1421	99.8	unpubl
JK19.3	Cardiac pacemaker	Japan	2017	LC341281	1349/1349	100	unpubl
*“P. humerusii”, n* = *1*							
R7A_C5_IA	Surface from ISS	Austria	2019	LR215132	1275/1277	99.8	unpubl
*Propionibacterium/Cutibacterium sp., n* = *7*							
Met-C3	Dental plaque	USA	2009	GQ422729	1484/1485	99.9	unpubl
P5998	Vagina	France	2018	LT996136	1486/1486	100	unpubl
F0672	Oral microbiome	USA	2018	CP017040	1486/1486	100	unpubl
KB17-24,694	Blood	Japan	2018	LC414574	1366/1366	100	unpubl
M12^T^	Meibomian glands	Japan	2019	LC466959	1483/1486^b^	99.8	Dekio et al. (2020)
NM47_B9-13	Murine gut	Canada	2019	MK929068	1486/1486	100	unpubl
602588-20-USB	Implant hip	Switzerland	2020	HG992826	1486/1486	100	Present study

Database analysis based on BLAST analysis with parameters: “nucleotide collection, exclude uncultured”; based on extracted *C. modestum* 16S rRNA gene sequence of M12^T^, accession no. BJEN0100000

^a^Main nucleotide differences at the end of the sequence indicating probable sequencing errors

^b^Strain M12^T^: 16S rRNA sequence shows 3 nucleotide differences compared with extracted 16S rRNA sequence of the genome

Genome sequences of six additional “*P. humerusii*” strains are present in public databases (Table 2), (Butler-Wu et al. 2011; Dekio et al. 2020). Table 2 also includes genome sequence data of our isolate 602588-20-USB, *C. modestum* strain M12^T^, and *C. acnes* ATCC 6919^T^ for comparison. 16S rRNA gene sequences extracted from the “*P. humerusii*”, 602588-20-USB and *C. modestum* M12^T^ genomes were 100% identical and dDDH and ANI values indicated that these organisms represented a single species, i.e. *C. modestum*. In Fig. 1, a whole genome SNP phylogenetic tree of the isolates listed in Table 2 is presented and reveales a high genomic homogeneity among all *C. modestum* isolates.

**Table 2:** List of available *C. modestum* genomes

Strain	Organism	Specimen	Country	Year	Accession no	16S rRNA gene		dDDH^a^	ANI^b^	References
						(bp)	(%)	(%)	(%)	
602588-20-USB	*C. modestum*	Implant hip	CH	2020	PRJEB41775USB					Present study
HL044PA1	«*P. humerusii*»	Skin	USA	2010	ADZU01000000	1486/1486	100	99.8	99.96	unpubl
HL037PA2	«*P. humerusii*»	Skin	USA	2010	ADYH01000000	1486/1486	100	99.7	99.95	unpubl
HL037PA3	«*P. humerusii*»	Skin	USA	2010	ADXV01000000	1486/1486	100	99.7	99.95	unpubl
P08	«*P. humerusii*»	Humerus	USA	2011	AFAM01000000	1486/1486	100	99.7	99.95	Butler-Wu et al. (2011)
P5998	«*P. humerusii*»	Vagina	F	2018	UWOQ01000000	1486/1486	100	98.1	99.73	unpubl
F0672	*Propionibacterium* sp.	Oral microbiome	USA	2018	CP017040	1486/1486	100	99.9	99.97	unpubl
T33958	«*P. humerusii*»	Skin shoulder	USA	2018	PCZR010000000	1486/1486	100	99.8	99.96	unpubl
M12^T^	*C. modestum*	Meibomian glands	J	2019	BJEN0100000	1486/1486	100	99.6	99.96	Dekio et al. (2020)
ATCC6919^T^	*C. acnes*	Facial acne	UK	2018	GCA_003030305	1459/1485	98.25	31.6	85.93	

Genomes related to *C. modestum* compared with genome of isolate 602588-20-USB. *C. acnes* is included as an outgroup

^**a**^Digital DNA-DNA hybridization formula 2 (Ref. Meier-Kolthoff et al. (2013))

^b^Two way average nucleotide identity (Ref. Goris et al. (2007))

In addition, we retrieved more than 100 sequence database entries of uncultured bacterium clone sequences from the human skin with 99.9–100% identity of a length of 1330 bp or longer compared to extracted *C. modestum* 16S rRNA sequence M12^T^. These sequences all belong to human skin microbiome data from three different studies (Grice et al. 2009; Kong et al. 2012; Oh et al. 2013).

## Genome data availability

Genome data is deposited in ENA project PRJEB41775 and the 16SrRNA gene sequence under accession no. HG992826.

## Discussion

We report a novel clinical isolate belonging to the recently described *C. modestum* (Dekio et al. 2020). Correct species identification was enabled only by partial 16S rRNA gene sequencing and whole genome analysis. Unfortunately, the 16S rRNA gene analysis was hampered by three incorrect nucleotides within the 16S rRNA reference sequence of *C. modestum* M12^T^ (accession no. LC466959), and incorrect *C. acnes* species designations in multiple 16S rRNA sequence entries.

The catalase test is an important biochemical characteristic for preliminary identification of *Cutibacterium* sp. Despite performing API Coryne analysis that comprises the catalase reaction, this result was not reported in the taxonomic proposal of *C. modestum* as a novel species (Dekio et al. 2020). The indole test is another basic biochemical reaction for biochemical identification of *Cutibacterium* sp. which was reported negative in the Dekio et al. study (Dekio et al. 2020). In contrast, our strain showed a clear positive indole and positive catalase reaction which confirms data reported for the tentatively characterised “*P. humerusii*” strain (Butler-Wu et al. 2011).

Routine MALDI-TOF MS failed to identify *C. modestum* because this species is not yet recorded in the commercial database. Dekio et al. (2020) reported four predominant MALDI-TOF MS peaks at 3493, 3712, 6986 and 7424 Da in *C. modestum* M12^T^. The same *m/z* peaks were also present in the spectrum of our isolate confirming their diagnostic value for future identification of *C. modestum* with MALDI-TOF MS (see supplementary materials figure S2).

The retrospective analysis of 16S rRNA sequence entries showed that *C. modestum* represents a bacterial organism of considerable clinical significance which often has been misidentified as *C. acnes*. Unfortunately, these incorrect data may lead to further misidentifications in current diagnostic applications based on 16S rRNA gene BLAST analysis.

Comparative analysis of the available genomes of this bacterium clearly indicates that *C. modestum* and “*P. humerusii*” represent the same species and consequently the “*P. humerusii*” database entries should be renamed (Table 2). Surprisingly, the corresponding phylogenetic tree only shows minimal genomic differences with exception of strain P5998 among the *C. modestum* and “*P. humerusii*” strains despite the origin of the isolates from different continents (Fig. 1).

We demonstrate for the first time that novel *C. modestum* might represent an organism of the normal skin microbiota. The high similarity to multiple uncultured clone sequences could indicate that this organism is difficult to detect using conventional cultural methods.

To conclude, the isolation and identification of *Cutibacterium* spp. remains challenging. The correct description of very recently named *C. modestum* and the availability of a correct 16S rRNA sequence of the type strain may help to clarify the taxonomical uncertainty concerning “*P. humerusii*”. *C. modestum* is identical to the previously named “*P. humerusii*” and represents a further clinically important species within the genus *Cutibacterium.*

## Supplementary Information

Below is the link to the electronic supplementary material.

Supplementary file1 (PDF 330 KB)

Supplementary file2 (PDF 253 KB)

## Data Availability

Sequences obtained in this study have been deposited in ENA Project PRJEB41775 and the 16S rRNA gene sequence is found under assession no. HG992826. The datasets used and/or analysed during the current study are available from the corresponding author on reasonable request.
