# Supplementary material for: Cutibacterium modestum and “Propionibacterium humerusii” represent the same species that is commonly misidentified as Cutibacterium acnes
Source: Antonie Van Leeuwenhoek. 2021 May 7;114(8):1315–20. doi: 10.1007/s10482-021-01589-5 (PMC8286925; doi:10.1007/s10482-021-01589-5)
Supplement: Supplementary file 1 — Supplementary file1 (PDF 330 KB) [file 10482_2021_1589_MOESM1_ESM.pdf]

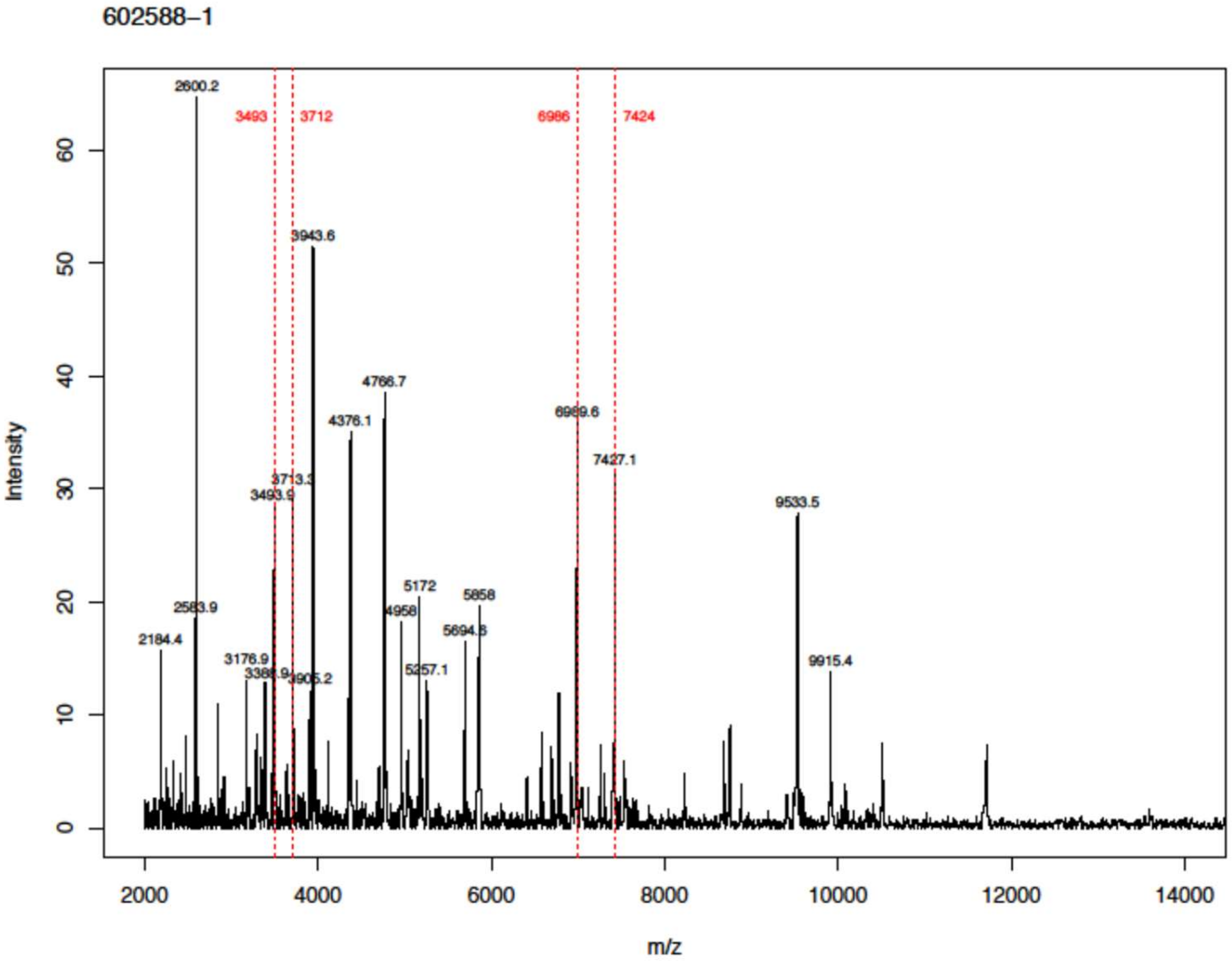

Supplementary material figure 1: Isolate 602588-20-USB; MALDI-TOF MS

Cutibacterium modestum* and *“Propionibacterium humerusii”* represent the same species that is commonly misidentified as *Cutibacterium acnes
Antonie van Leeuwenhoek
Daniel Goldenberger, Kirstine K Søgaard, Aline Cuénod, Helena Seth-Smith, Daniel de Menezes, Peter Vandamme, Adrian Egli
University Hospital Basel, Basel, Switzerland, E-Mail: daniel.goldenberger@usb.ch
