# Supplementary material for: Cutibacterium modestum and “Propionibacterium humerusii” represent the same species that is commonly misidentified as Cutibacterium acnes
Source: Antonie Van Leeuwenhoek. 2021 May 7;114(8):1315–20. doi: 10.1007/s10482-021-01589-5 (PMC8286925; doi:10.1007/s10482-021-01589-5)
Supplement: Supplementary file 2 — Supplementary file2 (PDF 253 KB) [file 10482_2021_1589_MOESM2_ESM.pdf]

Supplementary material table 1

Antimicrobial susceptibility results from *Cutibacterium modestum* strain 602588-20-USB. MIC, minimum inhibitory concentration. Cutibacteria are intrinsically-resistant to 5-nitroimidazole agents such as metronidazole and aminoglycosides such as gentamicin.

**Antimicrobial**	**MIC (mg/L)**
Penicillin	0.006
Amoxicillin	<0.016
Amoxicillin/clavulanic acid	<0.016
Piperacillin/tazobactam	0.064
Ceftriaxone	<0.016
Cefuroxime	<0.016
Cefepime	0.125
Imipenem	0.003
Erythromycin	0.032
Clindamycin	0.016
Tetracycline	0.125
Tigecycline	0.032
Ciprofloxacin	0.125
Moxifloxacin	0.125
Vancomycin	0.25
Gentamicin	6
Rifampicin	<0.002
Linezolid	0.25
Daptomycin	1
Metronidazole	>256

Cutibacterium modestum* and *“Propionibacterium humerusii”* represent the same species that is commonly misidentified as *Cutibacterium acnes

Antonie van Leeuwenhoek

Daniel Goldenberger, Kirstine K Søgaard, Aline Cuénod, Helena Seth-Smith, Daniel de Menezes, Peter Vandamme, Adrian Egli
University Hospital Basel, Basel, Switzerland, E-Mail: daniel.goldenberger@usb.ch
